# Enhanced Antitumor Activity by the Combination of Dasatinib and Selinexor in Chronic Myeloid Leukemia

**DOI:** 10.3390/ph17070894

**Published:** 2024-07-05

**Authors:** Mariarita Spampinato, Tatiana Zuppelli, Ilaria Dulcamare, Lucia Longhitano, Domenico Sambataro, Annalisa Santisi, Amer M. Alanazi, Ignazio A. Barbagallo, Nunzio Vicario, Rosalba Parenti, Alessandra Romano, Giuseppe Musumeci, Giovanni Li Volti, Giuseppe A. Palumbo, Francesco Di Raimondo, Anna Nicolosi, Sebastiano Giallongo, Vittorio Del Fabro

**Affiliations:** 1 Department of Biomedical and Biotechnological Sciences, Section of Biochemistry, University of Catania, 95123 Catania, Italy; mariaritaspampinato93@gmail.com (M.S.); tatiana.zuppelli@gmail.com (T.Z.); lucialonghitano2891@gmail.com (L.L.); ignazio.barbagallo@unict.it (I.A.B.); livolti@unict.it (G.L.V.); 2 Department of Clinical and Experimental Medicine, University of Catania, 95123 Catania, Italy; dulcamareilaria@gmail.com (I.D.); d.sambataro@hotmail.it (D.S.); 3 Department of Scienze Mediche Chirurgiche e Tecnologie Avanzate “G.F. Ingrassia”, University of Catania, 95123 Catania, Italy; annalisa_santisi@hotmail.it (A.S.); palumbo.gam@gmail.com (G.A.P.); 4 Pharmaceutical Biotechnology Laboratory, Department of Pharmaceutical Chemistry, College of Pharmacy, King Saud University, Riyadh 11451, Saudi Arabia; amalanazi@ksu.edu.sa; 5 Department of Biomedical and Biotechnological Sciences, Section of Physiology, University of Catania, 95123 Catania, Italy; nunzio.vicario@unict.it (N.V.); parenti@unict.it (R.P.); 6 Division of Hematology, Department of General Surgery and Medical-Surgical Specialties, A.O.U. “Policlinico-Vittorio Emanuele”, University of Catania, 95123 Catania, Italy; sandrina.romano@gmail.com (A.R.); diraimon@unict.it (F.D.R.); 7 Department of Biomedical and Biotechnological Sciences, Section of Anatomy, Histology and Movement Sciences, University of Catania, 95123 Catania, Italy; g.musumeci@unict.it; 8 Hospital Pharmacy Unit, Ospedale Cannizzaro, 95125 Catania, Italy; annanicolosi@hotmail.com; 9 Division of Hematology with BMT, A.O.U. Policlinico “G.Rodolico-San Marco”, 95123 Catania, Italy; vdelfabro@yahoo.it

**Keywords:** chronic myeloid leukemia, tyrosine kinase inhibitors, Selinexor, mitochondria

## Abstract

Background: Chronic myeloid leukemia is a hematological malignancy characterized by the abnormal proliferation of leukemic cells. Despite significant progress with tyrosine kinase inhibitors, such as Dasatinib, resistance remains a challenge. The aim of the present study was to investigate the potential of Selinexor, an Exportin-1 inhibitor, to improve TKI effectiveness on CML. Methods: Human CML cell lines (LAMA84 and K562) were treated with Selinexor, Dasatinib, or their combination. Apoptosis, mitochondrial membrane potential, and mitochondrial mass were assessed using flow cytometry. Real-time RT-PCR was used to evaluate the expression of genes related to mitochondrial function. Western blot and confocal microscopy examined PINK and heme oxygenase-1 (HO-1) protein levels. Results: Selinexor induced apoptosis and mitochondrial depolarization in CML cell lines, reducing cell viability. The Dasatinib/Selinexor combination further enhanced cytotoxicity, modified mitochondrial fitness, and downregulated HO-1 nuclear translocation, which has been associated with drug resistance in different models. Conclusions: In conclusion, this study suggests that Dasatinib/Selinexor could be a promising therapeutic strategy for CML, providing new insights for new targeted therapies.

## 1. Introduction

Chronic myeloid leukemia (CML) is a complex and multifaceted clonal hematopoietic stem cell disorder [1]. It is distinctively characterized by a substantial increase in both leukemic progenitors and mature cells, which can be observed in patients’ bone marrow (BM) and peripheral blood (PB) [2]. This increase is a hallmark of the disease and plays a critical role in its progression and manifestation. In this context, the development of tyrosine kinase inhibitors (TKIs) has fundamentally altered the treatment landscape of CML [1]. Among these, Imatinib, often hailed as the “gold standard”, and the second-generation Dasatinib have been pivotal in transforming CML into a manageable condition [3]. However, despite these advancements, the emergence of TKI resistance continues to present a formidable clinical challenge, underscoring the need for ongoing research and the development of new treatment strategies [4].

A central player in the pathophysiology of CML is the BCR-ABL fusion protein [1]. Predominantly localized in the cytoplasm, this protein is notorious for its interaction with a variety of signaling pathways, including, but not limited to, the MAPK, JAK-STAT, and PI3K/AKT pathways. These interactions are not only crucial in the disease’s progression but also in its response to various treatments [5]. Although TKIs have achieved significant success in managing CML, the medical community is constantly seeking to identify new vulnerabilities within the disease’s mechanism to enable sustained therapeutic responses and overcome the issue of drug resistance.

The microenvironment of CML plays a pivotal role in its pathology. Within this environment, there are intricate and dynamic interactions between various cellular components, including mesenchymal stromal cells, osteoblasts, osteoclasts, endothelial cells, and healthy hematopoietic stem cells (HSCs) [6]. These interactions are facilitated through a complex network of molecules and signaling pathways, which are critical for understanding both the progression of the disease and potential therapeutic targets.

Recent studies have highlighted that CML cells exhibit a marked increase in mitochondrial oxidative functions compared to normal HSCs [7,8]. This observation is particularly significant as mitochondrial oxidative metabolism is not only essential for energy production but also increasingly recognized as a key driver of metabolic plasticity in cancer cells [9]. This metabolic adaptation is thought to contribute to relapses following chemotherapy and the development of resistance to drugs.

Understanding these dynamics within the CML microenvironment provides valuable insights into potential therapeutic strategies. By targeting the unique metabolic requirements and interactions of CML cells, it may be possible to develop more effective treatments that can overcome drug resistance and prevent relapse.

In the realm of novel therapeutic strategies, targeting mitochondrial function has emerged as a particularly promising approach. A notable innovation in this domain is the development of Selinexor (XPOVIO™), which stands out as a first-of-its-kind, orally administered small-molecule inhibitor of Exportin-1 (XPO1). XPO1 plays a crucial role as a major nuclear exporter of a wide range of critical cellular components. These components include tumor-suppressor proteins, growth regulators, and oncoprotein mRNAs [10,11].

The inhibition of XPO1 by Selinexor leads to the accumulation of these vital proteins within the nucleus. This accumulation results in rapid cell cycle arrest and the induction of apoptosis, thereby effectively halting the progression of diseased cells [12]. Currently, Selinexor is in preregistration for various hematological diseases and has shown considerable promise in clinical trials. However, the specific role and potential development of Selinexor in the treatment of chronic myeloid leukemia (CML) remain areas ripe for further investigation and exploration.

In this comprehensive study, we aim to assess the effectiveness of Selinexor both as a monotherapy and in combination with Dasatinib in treating CML cell lines. Additionally, our research seeks to deepen our understanding of how Selinexor’s inhibition of XPO1 affects mitochondrial activity within CML cells. This is a critical area of investigation as it has the potential to unveil new vulnerabilities in CML that can be targeted pharmacologically. Through this, we hope to develop more precise and effective therapies for patients suffering from CML, addressing the existing challenges and paving the way for more successful treatment outcomes.

## 2. Results

### 2.1. Selinexor Induces Apoptosis in CML Cell Lines by Mitochondrial Depolarization

**Figure 1:**
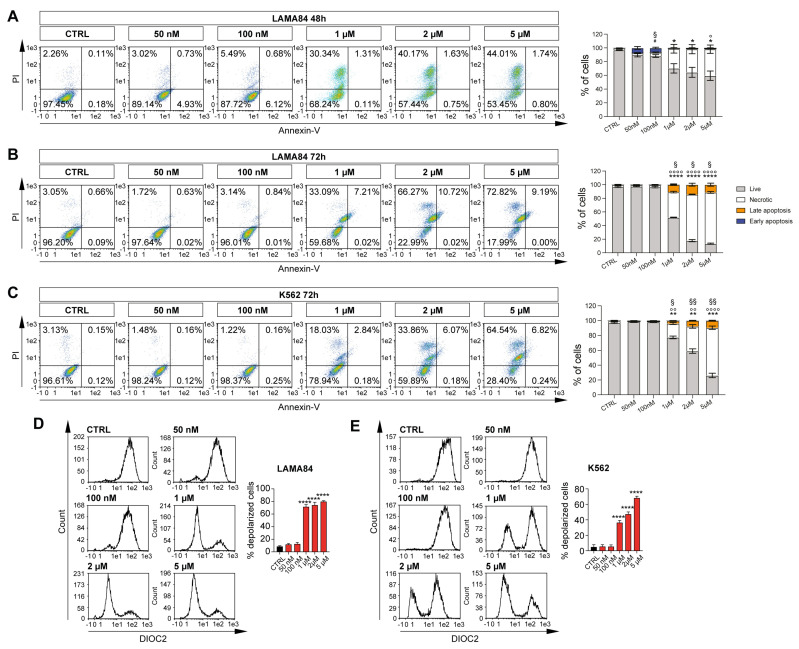
Selinexor elicits an apoptotic effect on CML cell lines. Representative flow cytometry graphs and relative quantitation of annexin V/PI assay on LAMA84 treated at 48 h (**A**) and 72 h (**B**) with dosages of 50 nM, 100 nM, 1 μM, 2 μM, and 5 μM. (**C**) Representative flow cytometry graphs and relative quantitation of annexin V/PI assay on K562 treated at 72 h with dosages of 50 nM, 100 nM, 1 μM, 2 μM, and 5 μM. (**D**) Flow cytometry analysis and relative quantitation of membrane depolarization assessment following 48 h of 50 nM, 100 nM, 1 μM, 2 μM, and 5 μM of Selinexor treatment on LAMA84. (**E**) Flow cytometry analysis and relative quantitation of membrane depolarization assessment following 72 h of 50 nM, 100 nM, 1 μM, 2 μM, and 5 μM of Selinexor treatment on K562. Data are reported as mean ± SD. Graphs are representative of 4 experimental replicates. * *p* < 0.05; ** *p* < 0.01; *** *p* < 0.001 and **** *p* < 0.0001 comparing live or depolarized cells to control. ° *p* < 0.05; °° *p* < 0.01; and °°°° *p* < 0.0001 comparing necrotic cells to control. # *p* < 0.05 comparing early apoptosis cells to control. § *p* < 0.05 and §§ *p* < 0.01 comparing late apoptosis cells to control.

Selinexor has been reported to exacerbate its therapeutic potential by triggering accumulating apoptotic events either alone or in combination [13]. In order to investigate its impact on CML, we, therefore, treated K562 and LAMA84 with increasing Selinexor doses (50 nM, 100 nM, 1 μM, 2 μM, and 5 μM). Interestingly, following 48 h of Selinexor treatment, LAMA84 showed a significant sensitivity to 1 μM, 2 μM, and 5 μM of Selinexor, eventually showing a 44%, 51%, and 56% decrease in viable cells compared to its control counterpart (*p* < 0.05; Figure 1A). At the same time, the 5 μM dosage also increased the percentage of necrotic cells (*p* < 0.05; Figure 1A). Of note, the increase in early and late apoptotic populations was also significant following 48 h of treatment with 100 nM of Selinexor (*p* < 0.05; Figure 1A). A similar trend, characterized by a decrease in viable cells, along with an increase in necrotic and late apoptotic populations at 1 μM, 2 μM, and 5 μM of Selinexor, was also mirrored 72 h post-treatment (*p* < 0.05 for late apoptosis cells; *p* < 0.0001; Figure 1B).

K562, on the other hand, shows a significant sensitivity to Selinexor only following 72 h of treatment, eventually displaying a 17%, 36%, and 48% reduction in viable cells in 1 μM, 2 μM, and 5 μM of treated cells, respectively (*p* < 0.01 and *p* < 0.001; Figure 1C). At the same time, an increase in necrotic (*p* < 0.001 and *p* < 0.0001) and late apoptotic (*p* < 0.05 and *p* < 0.01) populations was detected (Figure 1C). Interestingly, we did not observe any significant change in cell viability following 48 h of treatment on K562.

Selinexor has been reported to selectively inhibit the nuclear-to-cytoplasmic transport protein XPO1 [14]. Therefore, we sought to investigate mitochondrial depolarization following Selinexor supplementation in our CML in vitro cell model. Interestingly, our data on LAMA84 highlight a significant increase in mitochondrial depolarization upon 48 h of Selinexor treatment at 1 μM, 2 μM, and 5 μM, in turn, overlapping the annexin V/PI data (*p* < 0.0001; Figure 1D). In accordance with our previous results, K562 displayed Selinexor-induced mitochondrial depolarization only 72 h after treatment with 1 μM, 2 μM, and 5 μM in a dose-dependent manner (*p* < 0.0001; Figure 1E).

Overall, these data show that Selinexor-induced cell death in the CML in vitro cell model is mediated by mitochondrial depolarization.

### 2.2. Selinexor Increases Cytotoxicity of Dasatinib in LAMA84 Cell Line

**Figure 2:**
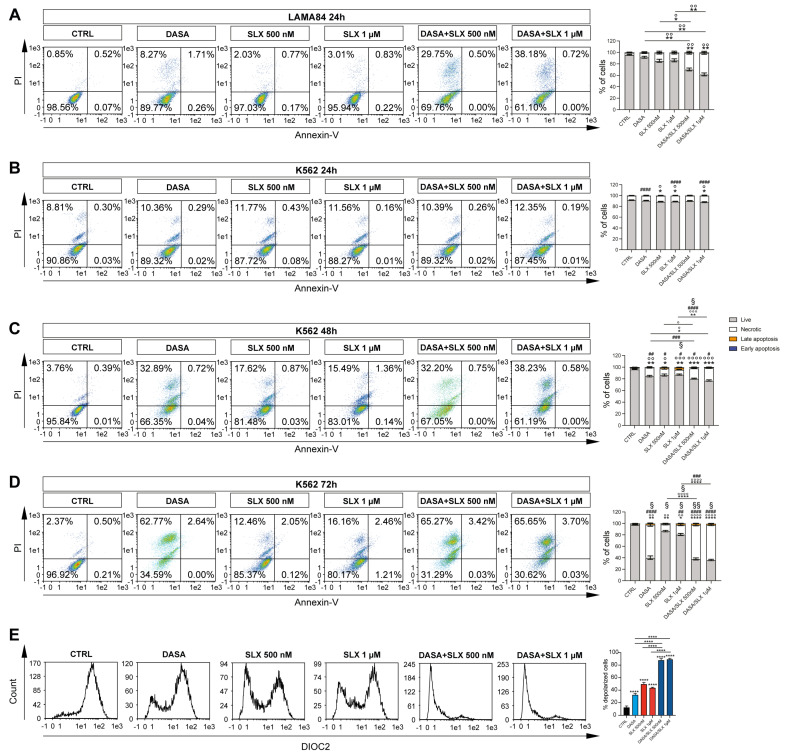
Selinexor increases Dasatinib’s effects on CML cell lines. (**A**) Representative flow cytometry graphs and relative quantitation of annexin V/PI assay on LAMA84 treated for 24 h with 2 nM of Dasatinib or 500 nM and 1 μM of Selinexor either alone or in combination. Representative flow cytometry graphs and relative quantitation of annexin V/PI assay on K562 treated for 24 h (**B**), 48 h (**C**), and 72 h (**D**) with 2 nM of Dasatinib or 500 nM and 1 μM of Selinexor either alone or in combination. (**E**) Flow cytometry analysis and relative quantitation of membrane depolarization assessment on LAMA84 following 48 h with 2 nM of Dasatinib or 500 nM and 1 μM of Selinexor either alone or in combination. Data are reported as mean ± SD. Graphs are representative of 4 experimental replicates. * *p* < 0.05; ** *p* < 0.01; *** *p* < 0.001; and **** *p* < 0.0001 comparing live or depolarized cells to control. ° *p* < 0.05; °° *p* < 0.01; °°° *p* < 0.001; and °°°° *p* < 0.0001 comparing necrotic cells to control. # *p* < 0.05; ## *p* < 0.01; ### *p* < 0.001; and #### *p* < 0.0001 comparing early apoptosis cells to control. § *p* < 0.05 and §§ *p* < 0.01 comparing late apoptosis cells to control.

Given the efficacy of treatment with Selinexor against the viability of CML cell lines and its probable involvement in the induction of mitochondrial damage, we sought to investigate a possible synergic strategy involving the co-treatment of Selinexor and Dasatinib, a second-generation TKI already shown to be effective in the long-term treatment of CML patients [15]. We, therefore, treated LAMA84 and K562 cell lines with 2 nM of Dasatinib alone or in combination with Selinexor (500 nM and 1 μM) for 24, 48, and 72 h, eventually performing annexin V/PI staining to assay their viability. Interestingly, our data display a marked decrease in LAMA84 viability following 24 h of the Dasatinib/Selinexor combination, either at 1 μM or 5 μM, in comparison to the experimental condition in which the drugs were used alone (*p* < 0.05; *p* < 0.01 Figure 2A). K562, on the other hand, shows no significant decrease in cell viability at this time point compared to treatment with single drugs (Figure 2B). However, the co-treatment exploited its synergic effect after 48 h of its administration (*p* < 0.05; *p* < 0.01; Figure 2C) and was enhanced at 72 h (*p* < 0.0001; Figure 2D) in comparison to its control counterpart and the experimental condition where drugs were used alone. In both time points, we also detected a marked increase in necrotic (*p* < 0.05; *p* < 0.001; *p* < 0.0001 Figure 2C,D), early (*p* < 0.001; Figure 2C,D), and late (*p* < 0.05 Figure 2C,D) apoptotic populations following Dasatinib/Selinexor treatment compared to its single drug counterparts.

Given these data, LAMA84 is significantly sensitive to Selinexor treatment, either alone or in combination with Dasatinib. We, therefore, decided to investigate the mechanisms underlying Selinexor-induced effects on this CML in vitro cell model. For this purpose, we assessed mitochondrial depolarization in LAMA84 treated for 24 h either with Dasatinib, Selinexor, or their combination (Figure 2E). Our data display a significant decrease in mitochondrial depolarization triggered by Dasatinib treatment compared to untreated cells (DioC2(3) MFI: 31.98 ± 0.02828427 versus 12.3 ± 0.42426407; *p* < 0.0001). Interestingly, Dasatinib/Selinexor treatment significantly increased the percentage of Dasatinib-induced depolarized cells by about 78% (*p* < 0.0001). Overall, these data show a significant synergic effect retained by Dasatinib/Selinexor against CML, which might be linked to increased mitochondrial depolarization caused by Selinexor supplementation.

### 2.3. Dasatinib/Selinexor Combination Decreases CML Mitochondrial Efficiency

**Figure 3:**
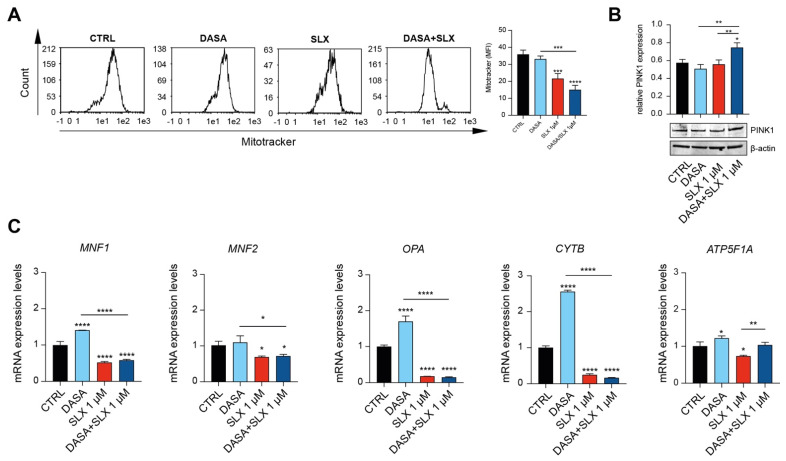
Selinexor impairs CML cell line mitochondrial dynamics. (**A**) Flow cytometry gating and relative quantitation of Mitotracker Red on LAMA84 treated for 48 h with 2 nM of Dasatinib or 1 μM of Selinexor either alone or in combination. (**B**) Western blot analysis and relative quantitation assessing PINK5 accumulation in LAMA84 following 48 h of treatment with 2 nM of Dasatinib or 1 μM of Selinexor either alone or in combination. (**C**) Real-time PCR analysis assessing MNF1, MNF2, OPA, CYTB, and ATP5F1A expression on LAMA84 following 48 h of treatment with 2 nM of Dasatinib or 1 μM of Selinexor either alone or in combination. Data are reported as mean ± SD. Graphs are representative of 4 experimental replicates. * *p* < 0.05; ** *p* < 0.01; *** *p* < 0.001; and **** *p* < 0.0001 compared to control or Dasatinib.

Our results suggest that Selinexor’s cytotoxic effect might be elicited through mitochondrial impairment. To further investigate this outcome, we, therefore, sought to assess the LAMA84 mitochondrial membrane potential using Mitotracker Red. Interestingly, compared to control cells and the experimental condition involving Dasatinib treatment, our flow–cytometry data unveil a significant decrease in mitochondrial membrane potential mediated by 24 h of Selinexor treatment (Figure 3A). Furthermore, we decided to assess PINK1 accumulation in LAMA84 treated with Dasatinib, Selinexor, or their combination. The PINK1/Parkin cascade is responsible for priming damaged mitochondria for mitophagy [16]. Interestingly, our data show a significant increase in the PINK1 level in the experimental condition where LAMA84 was co-treated with Dasatinib and Selinexor in comparison to the control and cells treated with Dasatinib or Selinexor alone (Figure 3B). Following this treatment, it seems that cells co-treated with Dasatinib and Selinexor encounter a decreased mitochondrial fitness. Given this outcome, we, therefore, sought to assess the expression of different genes involved in mitochondrial dynamics: mitofusin 1 (MNF1), mitofusin 2 (MNF2), OPA1 mitochondrial dynamin-like GTPase (OPA), Cytochrome B (CytB), and ATP Synthase F1 Subunit Alpha (ATP5F1A). Interestingly, following 24 h of Dasatinib/Selinexor treatment, we observed a marked decrease in the expression of MNF1, MNF2, OPA, CYTB, and ATP5F1A compared to the control counterpart and the conditions involving Dasatinib and Selinexor treatment alone (Figure 3C). Therefore, our data detect impairment in the mitochondrial dynamic following Dasatinib supplementation in CML cells.

### 2.4. Dasatinib/Selinexor Combination Decreases HO-1 Nuclear Translocation

**Figure 4:**
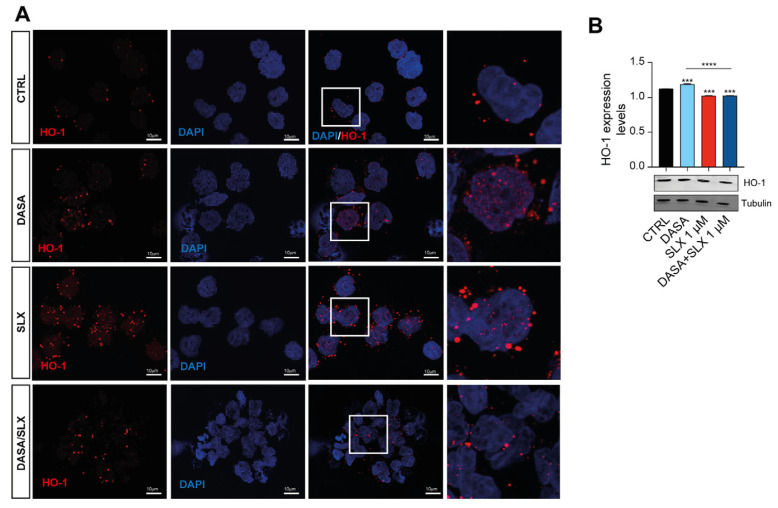
Selinexor decreases HO-1 nuclear translocation. (**A**) Immunofluorescence analysis (×20) investigating HO-1 nuclear localization on LAMA84 following 48 h of treatment with 2 nM of Dasatinib or 1 μM of Selinexor either alone or in combination. (**B**) Western blot analysis and relative quantitation detecting HO-1 accumulation in LAMA84 following 48 h of treatment with 2 nM of Dasatinib or 1 μM of Selinexor either alone or in combination. Data are reported as mean ± SD. Graphs are representative of 4 experimental replicates. *** *p* < 0.001 and **** *p* < 0.0001 compared to control or Dasatinib.

Co-treatment with Dasatinib and Selinexor showed a marked efficacy against Lama84, eventually showing a decreased viability after 24 h of treatment. In this context, HO-1 nuclear translocation elicits an antiapoptotic effect, therefore mediating TKI sensitivity [17]. In order to gain further insights into HO-1 involvement in the mechanisms mediated by Selinexor treatment, we assessed HO-1 dynamics in LAMA84 treated with Selinexor alone or in combination with Dasatinib. For this purpose, we first examined the levels of HO-1 protein by Western blot analysis after treatment with 2 nM of Dasatinib and 1 μM of Selinexor for 24 h. We found that Dasanitib treatment significantly increased HO-1 protein levels compared to untreated cells (*p* < 0.001, Figure 4A). Interestingly, Selinexor alone and in combination with Dasatinib significantly decreased HO-1 levels compared to Dasatinib (*p* < 0.0001) and control cells (*p* < 0.0001; Figure 4A). To further corroborate these data, we, therefore, performed a confocal microscopy analysis, which showed that HO-1 is localized in the cytoplasm of untreated LAMA84 cells (Figure 4B, top left panel). Interestingly, Dasatinib treatment triggers HO-1 nuclear translocation (Figure 4B, bottom left panel). The corroborating Western blot data and confocal microscopy show that Selinexor, either alone (Figure 4B, top right panel) or in combination with Dasatinib (Figure 4B, bottom right panel), decreased TKI-induced HO-1 nuclear translocation. Taken together, these data unveil a novel Selinexor-mediated effect on HO-1 cellular localization, possibly showing a strategy by which this molecule triggers cellular apoptosis in CML.

## 3. Discussion

CML is a clonal myeloproliferative disorder characterized by the presence of the Philadelphia chromosome, a result of the reciprocal translocation between chromosomes 9 and 22, leading to the BCR-ABL1 fusion gene. This fusion gene encodes for a constitutively active tyrosine kinase, which is central to the pathogenesis of CML. The advent of TKIs revolutionized the treatment of CML. TKIs, such as Imatinib, Dasatinib, and Nilotinib, specifically target the BCR-ABL1 oncoprotein, inhibiting its kinase activity, thereby reducing proliferation and promoting apoptosis in leukemic cells. Imatinib, the first TKI, demonstrated unprecedented success, transforming CML from a fatal disease to a manageable chronic condition for many patients. Despite this success, resistance and intolerance to TKIs remain significant challenges. Mutations within the BCR-ABL1 kinase domain, particularly the T315I mutation, are common mechanisms of resistance. Second-generation TKIs, like Dasatinib and Nilotinib, offer options for patients resistant or intolerant to Imatinib, and third-generation TKIs, such as Ponatinib, are effective against T315I mutations. The ongoing development of newer TKIs and combination therapies aims to overcome resistance, minimize side effects, and achieve deeper molecular responses, ultimately improving the prognosis and quality of life for CML patients [18]. However, long-term usage of these drugs, necessary to maintain remission, often leads to drug toxicity, acquired resistance, and substantial financial costs [4]. Additionally, approximately half of the patients experience rapid relapse after treatment discontinuation. This outcome is mainly related to leukemic stem cells (LSCs), a subpopulation of cancer cells within CML that possess the ability to self-renew and sustain the disease [19]. These cells are often resistant to conventional therapies, including TKIs, making them a critical target for achieving long-term remission and potential cure. In this context, recent research highlighted the importance of mitochondrial function in the survival and proliferation of LSCs. These cells are indeed characterized by an enhanced mitochondrial oxidative metabolism and are not only vital for energy production but also contribute to their metabolic plasticity and resistance to apoptosis [19,20,21].

However, there is a critical need to explore new therapeutic strategies [4]. For this purpose, our project initially investigated Selinexor’s cytotoxic effects on CML cell lines. Previous research showed that Selinexor selectively inhibits XPO1 by forming a slowly reversible covalent bond with cysteine 528 in the XPO1 cargo-binding pocket [11]. This inhibition leads to the accumulation of tumor-suppressor proteins (e.g., p53, p21, BRCA1/2, pRB, and FOXO) in the nucleus, reduces levels of oncoproteins (e.g., c-Myc, Bcl-xL, MDM2, and cyclins), induces cell cycle arrest, and promotes apoptosis [11]. Moreover, it disrupts the three-dimensional nuclear organization of telomeres in tumor cells while sparing normal cells [22]. Consistently, our study reveals that Selinexor treatment impacts the viability of CML cells and triggers mitochondrial membrane depolarization. This process is linked to the overexpression of pro-apoptotic proteins (BAX and BAK1) compared to antiapoptotic proteins (Bcl-1 and Bcl-2), likely due to XPO1 inhibition [23]. Given that primitive CML cells rely on heightened oxidative metabolism for survival, we then assessed the impact of a Dasatinib/Selinexor combination on mitochondrial status [7]. Our data highlight that XPO1 inhibition amplifies Dasatinib-induced apoptosis in LAMA-84 cells, accompanied by significant mitochondrial depolarization and a reduction in mitochondrial mass. Mitochondria play a pivotal role in maintaining cellular homeostasis through a dynamic process of fusion, fission, and mitophagy [24]. While cells treated with Dasatinib alone activate mitochondrial fusion and biogenesis as a protective response, Selinexor treatment interferes with this mechanism. This results in increased levels of the protein PINK1, which plays a key role in mitophagy [25]. Additionally, the Selinexor/Dasatinib combination reduces the expression of mitochondrial fusion-related genes, such as MNF1, MNF2, and OPA1, as well as ATP synthase, leading to pronounced mitochondrial impairment.

Furthermore, a HO-1 nuclear shift has recently emerged as a crucial mechanism through which BCR-ABL promotes cell survival, contributing to TKI resistance [26]. In agreement with this, Dasatinib treatment increases HO-1 levels and its nuclear translocation. In contrast, Dasatinib/Selinexor treatment significantly reduces HO-1 nuclear translocation, highlighting the impact of XPO1 inhibition on mitochondrial activity and HO-1 function. Of note, further studies, performed by generating a Dasatinib-resistant cell line would be needed to further assess the mechanism behind resistance acquisition in CML in vitro models.

## 4. Materials and Methods

### 4.1. Cell Culture

The human CML cell lines LAMA84 (CRL-3347) and K562 (CCL-243) were obtained from ATCC (Milan, Italy). The cells were cultured in RPMI 1640 (#11875093; ThermoFisher Scientific, Milan, Italy) supplemented with 10% fetal bovine serum (A5256701; ThermoFisher Scientific, Milan, Italy), 50 U/mL of penicillin, 50 µg/mL of streptomycin (#15070063; ThermoFisher Scientific, Milan, Italy), and 1% L-glutamine (#25030081; ThermoFisher Scientific, Milan, Italy) at 37 °C in 5% CO_2_. All the cell lines used were tested negative for mycoplasma and tested for the STR profile (Table 1).

### 4.2. Flow Cytometry

To evaluate apoptosis after drug treatment, cells were resuspended in PBS after centrifugation and stained with an annexin V FITC/7-ADD assay kit (IM3614; Beckman Coulter, Milan, Italy), as already published [27]. The apoptotic population was immediately evaluated by flow cytometry using a MACSQuant Analyzer 10 Flow Cytometer (Miltenyi Biotec, Bologna, Italy). The percentages of early apoptotic cells (annexin V+/7-ADD−) and late apoptotic cells (annexin V+/7-ADD+) were calculated and graphed. The membrane potential was evaluated, as previously described [27]. Briefly, 3,3-Diethylozacarbocyanine Iodide (DiOC2(3)) (D14730; ThermoFisher Scientific, Milan, Italy) was used to assess the mitochondrial membrane potential. For this purpose, the cells were incubated with 10 μM of DiOC2(3) for 30 min at 37 °C, washed twice, resuspended in PBS, and analyzed by flow cytometry through the detection of the green fluorescence intensity of DiOC2(3). In order to measure changes in the mitochondrial mass, 200 nM of a MitoTracker Red CMXRos probe (M46752; Thermo Fisher Scientific, Milan, Italy) for 30 min at 37 °C was used, as previously described [27]. After being washed twice, labeled mitochondria were analyzed by flow cytometry.

### 4.3. Real-Time RT-PCR

RNA extraction was performed, as previously described [28]. Reverse transcription was performed using a High-Capacity cDNA Reverse Transcription Kit (#4368814, Thermo Fisher Scientific, Milan, Italy). Then, the relative transcription of specific genes was determined by RT-qPCR using Brilliant III Ultra-Fast SYBR Green QPCR Master Mix (#600882, Agilent Technologies, Milan, Italy) and the 7900HT Fast Real-Time PCR System (Thermo Fisher). The expression of the following human genes involved in mitochondrial fitness maintenance was evaluated:

OPA1 mitochondrial dynamin, like GTPase (OPA1) (Fw: GTGCTGCCCGCCTAGAAA; Rw: TGACAGGCACC CGTACTCAGT); mitochondrial dynamics: mitofusin1 (MNF1) (Fw GGCATCTGTGGCCGAGTT; Rw: ATTATGCTAAGTCTCCGCTCCAA); mitofusin 2 (MNF2) (Fw: GCTCGGAGG CACATGAAAGT; Rw: ATCACGGTGCT CTTCCCATT); ATP Synthase F1 Subunit Alpha (ATP-synthase) (Fw: AGCTCAGCTC TTACTGCGG; Rw: GGTGGTAGT CCCTCATCAAACT); Cytochrome B (CytB) (Fw: TCCTCCCGTGAGGCCAAATATCAT; Rw: AAAGAATCGTGTGAGGGTGGGACT); Beta-2-microglobulin (B2M) (Fw: AGCAGCATCA TGGAGGTTTG; Rw: AGCCCTCCTA GAGCTACCTG); and glyceraldehyde-3-phosphate dehydrogenase (GAPDH) (Fw: AATGGGCAGC CGTTAGGAAA; Rw: GCCCAATAC GACCAAATCAGAG). For each sample, the relative expression level of the mRNA of interest was determined by comparison with the control housekeeping genes B2M and GAPDH using the 2^−∆∆Ct^ method.

### 4.4. Western Blot Analysis

Western blot analysis was performed, as previously described [29]. Briefly, for Western blot analysis, 10 μg of protein was loaded onto an 8% polyacrylamide gel Mini-PROTEAN TGXTM (#4561021; BIO-RAD, Milan, Italy), followed by electrotransfer to a nitrocellulose membrane Trans-Blot TurboTM (#1704150; BIO-RAD, Milan, Italy) using a Trans-Blot SE Semi-Dry Transfer Cell (#1703940; BIO-RAD, Milan, Italy). Subsequently, the membrane was blocked in Odyssey Blocking Buffer (927-70001; Licor, Milan, Italy) for 1 h at room temperature. After blocking, the membrane was washed three times in PBS for 5 min and incubated with rabbit anti-PINK1 (1:1000), mouse anti-HO-1 (1:1000), and mouse anti-β-actin (1:2000) (ab23707, ab13248, and ab8226, Abcam, Milan, Italy), overnight at 4 °C. The next day, the membranes were washed three times in PBS for 5 min and incubated with infrared anti-mouse IRDye800CW (1:5000) and anti-rabbit IRDye700CW secondary antibodies (1:5000) (Licor, Milan, Italy) in PBS/0.5% Tween-20 for 1 h at room temperature. All antibodies were diluted in Odyssey Blocking Buffer. The blots were visualized using an Odyssey Infrared Imaging Scanner (Licor, Milan, Italy), and the protein levels were quantified by the densitometric analysis of antibody responses. Data were normalized to protein levels of β-actin.

### 4.5. Immunofluorescence

An immunofluorescence assay was performed, as previously reported [29]. Briefly, after drug treatment, cells were pulled down on slides by cytospin and subsequently fixed with 4% formaldehyde for 20 min at room temperature. After three washings in PBS for 5 min, cells were fixed using 4% paraformaldehyde, permeabilized using 0.1% Triton X, and blocked to prevent nonspecific antibody binding using 2% bovine serum albumin. The slides were then incubated overnight at 4 °C with mouse anti-HO-1 (1:100) (anti-mouse; ab13248, Abcam, Milan, Italy). The next day, cells were washed three times in PBS for 5 min and incubated with goat anti-mouse FITC (A16067; Thermo Fisher Scientific, Milan, Italy) at 1:200 dilution for 1 h at room temperature. The slides were mounted with the medium containing DAPI (4,6-diamidino-2-phenylindole) to visualize nuclei. The fluorescent images were obtained using a Zeiss Axio Imager Z1 Microscope with the Apotome 2 system (Zeiss, Milan, Italy).

### 4.6. Statistical Analysis

Statistical analyses were conducted with Prism Software, version 9.0 (Graphpad Software Inc., La Jolla, CA, USA, RRID: rid_000081). Data were expressed as mean ± SD. Statistical analysis was carried out by one-way ANOVA, comparing the mean of each column with the mean of the Control, Dasatinib, and/or Selinexor groups, where needed. The multiple comparison was corrected using Tukey statistical hypothesis testing. A *p*-value of 0.05 was considered to indicate a statistically significant difference between the experimental and control groups.

## 5. Conclusions

The current therapeutic approach for chronic myeloid leukemia (CML) lacks a valuable strategy for patients displaying tumor relapse. To address this gap, we describe the usage of Selinexor, an XPO1 inhibitor, as a promising therapeutic strategy in CML treatment. Our data suggest that the synergistic activity of Selinexor and Dasatinib may rely on the induction of mitochondrial membrane depolarization, which ultimately impairs the viability of CML cells in vitro. Furthermore, the unique dependence on mitochondrial function distinguishes LSCs from normal hematopoietic stem cells and bulk leukemic cells, eventually presenting a therapeutic opportunity. Targeting mitochondrial pathways, for instance, by Dasatinib/Selinexor administration, might represent a possible strategy in selectively eradicating LSCs. In particular, by impairing mitochondrial function, it is possible to sensitize LSCs to apoptosis and reduce their ability to sustain the disease, potentially overcoming resistance and achieving more durable treatment responses in CML patients.

In conclusion, further investigations are warranted to delve deeper into the potential molecular and metabolic pathways activated by the Dasatinib/Selinexor combination. This exploration aims to identify specific vulnerabilities that can be targeted pharmacologically, thereby paving the way for more precise and effective treatments for CML.

## Figures and Tables

**Table 1:** STR profile of CML in vitro cell models.

LAMA84	K562
Amelogenin: X	Amelogenin: X
CSF1P0: 11, 12	CSF1P0: 9, 10
D13S317: 11, 12	D13S317: 8
D16S539: 11	D16S539: 11, 12
D5S818: 11, 12	D5S818: 11, 12
D7S820: 11	D7S820: 9, 11
THO1: 6, 7	THO1: 9.3
TPOX: 10, 11	TPOX: 8, 9
vWA: 14, 17	vWA: 16

## Data Availability

The original contributions presented in the study are included in the article, further inquiries can be directed to the corresponding author.
